# Draft Genome Sequences of Endophytic Isolates of Klebsiella variicola and Klebsiella pneumoniae Obtained from the Same Sugarcane Plant

**DOI:** 10.1128/genomeA.00147-18

**Published:** 2018-03-22

**Authors:** Fernando Reyna-Flores, Humberto Barrios-Camacho, Edgar Dantán-González, José Augusto Ramírez-Trujillo, Luis Fernando Lozano Aguirre Beltrán, Nadia Rodríguez-Medina, Ulises Garza-Ramos, Ramón Suárez-Rodríguez

**Affiliations:** a Laboratorio de Resistencia Bacteriana y Genómica de Bacterias, Centro de Investigación Sobre Enfermedades Infecciosas (CISEI), Instituto Nacional de Salud Pública (INSP), Cuernavaca, Morelos, Mexico; b Laboratorio de Estudios Ecogenómicos, Centro de Investigación en Biotecnología (CEIB), Universidad Autónoma del Estado de Morelos, Cuernavaca, Morelos, Mexico; c Laboratorio de Fisiología Molecular de Plantas, Centro de Investigación en Biotecnología (CEIB), Universidad Autónoma del Estado de Morelos, Cuernavaca, Morelos, Mexico; d Centro de Ciencias Genómicas, Programa de Genómica Evolutiva, Universidad Nacional Autónoma de México, Cuernavaca, Morelos, Mexico

## Abstract

Endophytic Klebsiella variicola KvMx2 and Klebsiella pneumoniae KpMx1 isolates obtained from the same sugarcane stem were used for whole-genome sequencing. The genomes revealed clear differences in essential genes for plant growth, development, and detoxification, as well as nitrogen fixation, catalases, cellulases, and shared virulence factors described in the K. pneumoniae pathogen.

## GENOME ANNOUNCEMENT

Klebsiella variicola has been described as an endophyte and has been identified in different plants (1). However, Klebsiella pneumoniae is considered a pathogen in humans and equally as an endophytic bacterium; nevertheless, few data and scarce genomes have been described for isolates obtained from plants. In the case of K. pneumoniae 342, obtained from maize (2), the isolate actually corresponds to K. variicola (3). A clear difference between these species is the capacity of K. variicola to fix atmospheric nitrogen and promote plant growth (1, 3–6). For this study, Klebsiella species colonies were obtained from the root of a sugarcane plant (ATMEX 96-40) grown in Cuautla, Morelos, Mexico. A multiplex PCR (M-PCR-1) (7) was used for the proper identification and differentiation of K. variicola and K. pneumoniae isolates. Colonies of both the K. variicola KvMx2 and K. pneumoniae KpMx1 isolates were identified and then used for whole-genome sequencing.

Total genomic DNA was extracted and purified using the DNeasy kit (Qiagen, Germany). Whole-genome sequences were generated using an Illumina (MiSeq) platform, and a total of 4,706,652 paired-end reads in K. variicola KvMx2 and 2,562,748 paired-end reads in K. pneumoniae KpMx1 with a length of 150 bp were obtained. Quality-based trimming was performed with the SolexaQA software, and *de novo* assembly was done with SPAdes version 3.1.1. The contigs were subjected to a scaffolding process with SSPACE version 2.0. For K. variicola KvMx2 and K. pneumoniae KpMx1, respectively, a total of 37 contigs (*N*_50_, 397,617 bp) and 50 contigs (*N*_50_, 377,295 bp) and estimated genome sizes of 5,528,301 bp (128× coverage) and 5,371,602 bp (72× coverage) were determined. Gene prediction and annotation were carried out using the bioinformatic MicroScope platform (8). In the K. variicola KvMx2 genome, a total of 5,431 coding DNA sequences (CDSs), 75 tRNA genes, and 6 rRNAs were found. In the K. pneumoniae KpMx1 genome, a total of 5,324 CDSs, 82 tRNA genes, and 9 rRNAs were found. The average G+C contents were similar in the two genomes, at 57.39% for K. variicola KvMx2 and 57.46% for K. pneumoniae KpMx1. An average nucleotide identity (ANI) >95% (9) confirmed the bacterial species.

A genomic comparison between K. variicola KvMx2 and K. pneumoniae KpMx1 showed a pangenome of 6,306 families of proteins, with a core genome of 4,306 families and a variable genome of 1,078 families of proteins for K. variicola KvMx2 and 922 families of proteins for K. pneumoniae KpMx1. The K. variicola KvMx2 genome revealed genes essential for the biosynthesis of indole-3-acetic acid (IAA), acetoin and 2,3-butanediol, and rhodanase, which are involved in plant growth, development, and detoxification of cyanide compounds (10), respectively. Likewise, the *K. variicola* genome contains the *nifJ-NifQ* nitrogen fixation gene cluster and is a clear difference between the two bacterial species. Otherwise, the catalase genes *bglX* and *katGE* are contained in both bacterial species; however, the cellulase *bglH* gene is contained only in K. variicola KvMx2.

With respect to virulence factors, the *entB*, *mrkABCDFHJL*, *ureA*, *uge*, and *wabG* genes are contained in both the K. variicola KvMx2 and K. pneumoniae KpMx1 genomes. Only in the K. variicola KvMx2 genome was the *kfuABC* operon identified. Finally, K. pneumoniae KpMx1 contained the copper resistance *pcoABCDER* operon.

### Accession number(s).

The annotated genome sequences are available at the European Nucleotide Archive under accession numbers FLLH01000001 to FLLH01000037 (K. variicola KvMx2) and FLLB01000001 to FLLB01000050 (K. pneumoniae KpMx1).
